# Professor Scarpa on Scirrhus and Cancer

**Published:** 1823-06-01

**Authors:** 


*Remarks and Practical Results of Observation on Scirrhus and Cancer.* By ANTONIO SCARPA, Emeritus Professor and Director of the Faculty of Medicine in the Royal Imperial University of Pavia, Chevalier of the Royal Order of the Iron Crown, Member of the Royal Academy of Sciences in Paris, F.R.S. &c. &c. Translated from the Italian. With an Appendix, containing an Account of a Sanguineous Tumour of the Breast, and some Annotations on the Text. By JAMES BRIGGS, Surgeon to the Public Dispensary, and Assistant Surgeon to the Lock-Hospital, 8vo. sewed, pp. 61, with a plate, London, 1823.

MR. BRIGGS informs us, in his preface, that the original of the following Translation appeared lately in the Transactions of the Imperial Institute of Milan, and may not be undeserving of the English surgeon's perusal. In it, he thinks, the doctrine of cancer being a local disease, in its incipient state, and consequently curable by early extirpation, is based on a fuller and more satisfactory body of evidence than has yet been laid before the public. Any thing indeed from the pen of the venerable and experienced Scarpa bears a passport to professional attention at least, if not esteem; and under this impression, we deem it our duty to give extended publicity to the celebrated Pavian Professor's observations.

Many have been the remedies proposed, as Mr. Briggs observes, for the dispersion of scirrhus and healing of cancer, but none of them have realized expectation. How indeed can we reasonably look for the restitution, by internal remedies, of a part, the organical structure of which is found to be wholly subverted, and, as it were, disjointed from the general system? It must be evident that no other means are left than the absolute removal or destruction of the part by a mechanical operation.

"Perdit medicina sæpe egrotos, quos chirurgia servare poterat."

The determination of the period when the diseased part may be removed with full assurance of success, and the sources of the frequent failure of the operation, are the principal objects of the present memoir.

The Professor commences by noticing the discrepancies of opinion, among men too of erudition and experience, whether scirrhus and cancer be *purely local*, or general and local affections—whether scirrhus attacks, without exception, every organic texture—whether it may be distinguished from other glandular tumours bearing an affinity to it—whether cancer manifest the same degree of malignancy in all structures—and finally, *why* the legitimate glandular cancer so constantly baffles the hand of the operator, and the action of the most powerful caustics and cauteries ?

The Professor thinks that we are enabled now to distinguish real scirrhus from other chronic tumours apparently similar to it—and to demonstrate in the most satisfactory manner the advantage arising from a preventive and timely recourse to manual operation.

"It appears, in the first place, from almost innumerable practical observations, that scirrhus and consequently cancer never attack primarily the lymphatic system, nor of necessity the absorbent glands. One or two examples adduced to the contrary, in which a supposed scirrhus had been extirpated with some of the lymphatic glands of the neck (for every chronic glandular tumor rather harder than common, even by surgeons of eminence, has been too hastily denominated such), have not been hitherto authenticated, much less confirmed, by rational experience.

The whole, or at least certainly some of the most remarkable of the *mucous* glands, as the *sublingual* and the *tonsillary*, are likewise known to be exempt from a primitive scirrhous and cancerous taint. With regard to the latter of these, the large hard tonsil improperly termed scirrhous, every experienced surgeon is aware that it may be removed, portion after portion, and even gradually destroyed, by the repeated applications of caustic, without any fear of its degenerating into cancer by such means. The *thyroid* gland, whether we are disposed to regard it as a lymphatic or mucous gland, never becomes *primarily* scirrhous or cancerous. And if in any instance this glandular body has been found eroded by cancerous ulceration, it has only been in consequence of its contiguity with the trachea or œsophagus affected by that disorder. Whenever consequently one or more of the lymphatic or any of the mucous glands already mentioned are found to be infected with cancer, it takes place only by the cancerous ichor being conveyed to them by means of the absorbent vessels, or by the infiltration of the morbid poison within the cellular tissue connecting the lymphatic gland with the cancerous part." 3.

In the next place, our author avers, that scirrhus and cancer are never met with *primarily* in the viscera, strictly so called, "with the exception of those internal parts which are furnished with an intimate covering of the reflected skin, as the larynx, œsophagus, stomach, rectum, vagina, and neck of the uterus." The indurations of the brain, fungi of the dura mater, chronic tubercles of the lungs, of the liver, spleen, omentum, pancreas, kidneys, ovaria, prostate, bladder, are in no respect, says our author, either scirrhous or cancerous, or of the nature of *medullary* fungi.* These chronic indolent tumours he thinks, or fungous tubercles of the viscera (provided they are not manifest consequences of general cancerous contamination) are, strictly speaking, " but morbid phenomena of general *scrophulous* diathesis, or else indurations either referable to previous acute inflammation imperfectly discussed, or produced by slow and long protracted inflammatory action."

Again, our author affirms that scirrhus and cancer never make their appearance before puberty, and seldom before the 25th year in either sex—the slow indurations of the mammæ in female children, the chronic tumefaction of the conglomerate glands, and the indolent enlargement of the testes in boys, being " invariably, and without exception, of scrofulous origin and character."

" In the fourth place, observation and experience teach us that cancer is never formed but in consequence of legitimate scirrhus affecting some of the external conglomerate glands, or of rigid indurated porri warts or malignant tubercles of the external or reflected skin partaking of the nature of scirrhus. Under one or other of these two forms the disease may make its appearance; and the ulceration which ensues upon it is that which is strictly cancerous; for an unclean fungous spreading sore with hard inverted edges, is a thing widely different from a malignant ulcer formed upon a part which had been previously indurated by genuine scirrhus." 6.

Conformably to our existing pathological knowledge, observes Professor Scarpa, there are but two organic textures, which serve as a nidus (if he may be allowed the expression) for the formation and development of scirrhus and cancer, viz. the external conglomerate glands and the skin. Among the former, the mammæ is most subject to the disease, next

---

* " Induration of the pancreas" says he, " is not unfrequent; but even in this morbid state, it preserves its natural internal conformation, that is, the lobular structure; which is not found to be the case in the substance of the scirrhous gland, the whole of which has been changed into an uniform lardaceous mass.

" The internal structure of tumors of the ovaria, is not dissimilar to that of *strumous* or *steatomatous* tumours, which is widely different from that of scirrhus.

" No surgeon has yet seen a genuine scirrhus, much less a cancer of the prostate gland. The lacerations which are sometimes made by the introduction of the catheter across it, are never found to degenerate into cancerous ulceration.

" If in any instance the neck of the urinary bladder has been found infected by cancer, it has only been in consequence of its connexion with the rectum or neck of the cancerous uterus." 4.

the parotid, maxillary, and lachrymal glands, together with the body of the testicle.

The latter of the two organic textures liable to scirrhus and cancer is the skin. In consequence of peculiarity of structure, the scirrhus presents itself in this tissue either in the form of a rigid wart, hard tubercle, or dark varicose knot.

"On an attentive examination, however, of the minute structure of these malignant tubercles, whether of the external or reflected skin, notwithstanding this diversity of external configuration, their internal substance is found to bear a close resemblance to the intimate, firm, *lardaceous* texture of the glandular scirrhus, intersected in like manner by whitish lines, and filled, like that of the scirrhus, with a dense, coagulated, viscid, albuminous fluid. And it is deserving of remark, with regard to these malignant scirrhi of the skin, that their destructive tendency is not manifested with an equal degree of virulence in every part of the integument. For, it is observed, that the real nature of these excrescences, is more destructive in proportion to the vascularity, sensibility, or important offices of the parts which are covered by the affected skin; cancer of the face and lips being less formidable than that of the internal nostrils, of the tongue, lachrymal caruncle, termed the *malignant encanthis*, that of the glans of the penis, of the rectum, of the vagina, or neck of the uterus." 8.

In respect to the last-mentioned part, our author has no doubt, from numerous anatomico-pathological examinations, that the primordia of cancer uteri are to be invariably traced to the ulceration of one or more of these small scirrhi, under the form of warts or tubercles, which are formed upon the reflection of skin investing the upper part of the vagina, and the orifice and neck of the uterus.

"One or more of these malignant hard tubercles upon the reflection of skin, increasing in volume, encircle the os uteri like a ring, and occasion the opening and morbid dilatation of this natural orifice, the edges of which become hard and irregular, and afterwards exulcerate; and, from being indolent as at first, become painful, giving sudden transient shootings; and presenting, when examined by the finger, excavations and irregular elevated edges around the orifice of the uterus, as well as at the upper part of the vagina, from which is discharged a thin bloody ichorous fluid, of a *lixivial* odour. The cancerous ulceration at first spreads superficially upon the neck of the uterus precisely as the cancer of the skin affecting the face; but afterwards penetrates more deeply, first destroying the substance of the cervix uteri, then that of its body, and, lastly, its fundus. All other chronic hard indolent tumors which originate from the internal or external surface of the body, or fundus of the uterus, when examined anatomically, present nothing in their minute structure which is in common with the true scirrhus and cancer; nor is there, I may venture to assert, in the whole records of surgery, any well-authenticated fact of cancer of the uterus having originated from any other part of this viscus, except from the skin reflected over the orifice of it, and investing the upper part of the vagina." 10.

We are not able to follow our author minutely in his diagnosis between scrophulous and scirrhous enlargements. The scrophulous diathesis and character are well delineated, and are but too familiar to the English practitioner. We shall extract a passage from our able author relative to scirrhus, as contrasted with scrophula.

"The peculiar and distinguishing symptoms of schirrhus are of a perfectly opposite character to those of struma affecting any of the external conglomerate glands. The scirrhus attacks persons of advanced age, of rigid fibre, and of a sanguineo-bilious temperament, in whom, if there be any suspicion of general vice of the habit, it is not that of scrophula. The scirrhus is *solitary*, that is, it affects only some one of the external conglomerate glands, and it rarely or ever happens that two really legitimate scirrhi are met with in the same individual. The scirrhus, from its first appearance, possesses an excessive and almost stony hardness, is perfectly indolent, in consequence of its not being associated, like the scrophulous or strumous swelling, with low chronic phlogosis. The scirrhus increases slowly in every direction, and feels to the touch as if composed of so many pieces of hard substance agglutinated to each other. Its insensibility, notwithstanding the increase of the tumor, is preserved, until it degenerates into cancer. This distinguishing character of the scirrhus, contrasted with those of struma, which, as I have stated, is not altogether insensible, has been remarked by Galen, in the following words:—"*Exquisitus scirrhus tumor est præter naturam sensu carens et durus: non exquisitus autem* (alluding to struma) *non omnino sine sensu est, sed ægre tamen admodum sentit.*"—The inveterate scirrhus, which is generally tuberculated, raises the skin irregularly, to which, in some parts, it forms an adhesion. As soon as the darting pains commence, the scirrhus, so far from encreasing in size, shrinks and acquires a degree of hardness approaching, one might say, to dryness, drawing inward along with it that portion of skin to which it had formed adhesion; precisely contrary to what happens in struma when the tumor has arrived at its largest bulk, and is on the point of bursting, or suppuration taking place within the substance of it.

"In regard to the difference between the internal altered texture of the strumous conglomerate gland and that of scirrhus, the following are the results, furnished by anatomical investigation. A coloured size-injection thrown into the arterial vessels of the strumous gland penetrates it at first very freely, but becomes soon effused into it, in consequence of the flaccidity and friability of the vessels which supply the substance of the gland. When a section is made of the strumous gland, a vascular compact substance presents itself, filled with a glutinous *(albuminous)* fluid, mixed with a granulous, sebaceous, or cretaceous substance. Between the body of the tumor and its exterior involucrum, there are always some vestiges of *coagu-*

*lable* lymph; and frequently also, in the internal part of it, indubitable proof of its having suffered inflammation, though slight and passive. In the scirrhus tumor, on the contrary, the injection, though of the finest kind, never fills but the principal arterial trunks of the diseased gland. The hardness of the substance composing the scirrhus is altogether peculiar to this tumor, and cannot be confounded even by the most inattentive observer with that of any other kind of chronic glandular enlargement. It has the appearance of softened cartilage, and strongly resembles the mollified substance of the cartilages and ligaments of the joints, in the change observed in the *white* swelling. When the scirrhus is divided through the centre, it presents a whitish surface, equally striped with bands still whiter than itself, like rays drawn from the centre to the circumference, or in the form of ramifications.* On pressing upon it, a transparent gelatinous (albuminous) fluid issues from it, which, diffusing itself upon the cut surface, renders it in a short time lucid, and as if it were covered by varnish. Lastly, if the struma and scirrhus are macerated together in water for a considerable length of time, the struma is found to separate into a soft spongy fimbriated mass, while the scirrhus retains nearly the peculiar hardness which it possessed before it was subjected to that process. This remarkable difference in the cohesion between the component parts of the scirrhus and those of struma, points out the reason why in some supposed cases of *occult* cancer, but which in reality were of a strumous nature, cavities have been found containing quantities of pure or bloody serum of two, four, and even six pounds weight, which certainly could never take place in the intimate hard tenacious substance of scirrhus." 19.

The real nature of warts and malignant tubercles of the external or reflected skin, is to be judged of from their unusual rigidity and hardness—from their being observed to be deprived of their natural tegument—from the unusual size and depth of the base, which seems to extend beyond the substance of the skin—from their being of a yellowish, livid, or dark colour, surrounded by a small red circle—from their rapid growth—from the intolerable pruritus excited by them, from which there is a slight yellow sanguinolent acrid serum, preceded by sudden darting pains.

In respect to the diagnosis between scirrhus and the incipient malignant medullary fungus, our author thinks it proper to state that the *former* attacks only some of the external conglomerate glands, and the exterior or reflected skin; while the medullary fungus has its origin in the sub-cutaneous or intermuscular cellular texture, or, as some suppose, even in the sheaths and substance of the nerves themselves.

---

* Baillie's Morbid Anatomy.—Abernethy's Surgical Works, vol. ii.

"The recent medullary fungus also, provided it is not too deeply seated, has conjoined with its hardness so remarkable a degree of *elasticity*, that this latter might be said to be the peculiar and characteristic symptom of this swelling, by which it is principally distinguished from the congenital varicose sanguineous tumor (hematodes.) The inveterate medullary fungus, which is in general much more extended than elevated, is partly hard and elastic, partly soft, with an apparently subjacent fluctuation; circumstances that are never met with in the scirrhus before it has degenerated into the state of occult cancer." 20.

We find it impossible to follow our author through his lucubrations on the constitutional generation of the scirrhous taint, and its deposition *locally* afterwards; but the following passage will convey a pretty clear idea of Professor Scarpa's doctrine on the point.

"If, therefore, during the first period of the scirrhus, the person affected by it enjoy general good health, and if, in the second stage of the disease, the symptoms of cancerous cachexy appear, it seems to be highly probable, from what has been already stated, that the scirrhus is originally and during its first period but the *deposition* of a malignant germ, generated in the system at large, and afterwards impelled by the vital forces, and concentrated wholly in the sinus of some of the external conglomerate glands, or upon some portion of external or reflected skin, where it is retained in a *quiescent* and *innocuous* state; and of consequence that the cancer is but the result of a local process of imperfect suppuration, excited in the intimate substance of the scirrhous gland, by which the malignant deposit is converted from this latent and inert state into cancerous ichor." 35.

From these premises, our author thinks it follows, as a necessary consequence, that the demolition of the scirrhus can never be attended with success, "unless the operation have been executed before the development of the latent morbid seed in the substance of the scirrhous gland, or malignant wart or tubercle of the skin—that is to say, *before the commencement of the lancinating pains*, and infection of the lymphatic glands connected with the seat of the occult cancer." This, we confess, is melancholy doctrine.*

In illustration of the great advantage of early extirpation, (the jet indeed of the whole publication,) our author adduces the testimony of Flagani, who only was unsuccessful in two cases out of twenty-seven, "performed within the first months from the appearance of the scirrhous tumour." The following will convey to the English reader the result of the Italian professor's experience.

---

* See also Mr. Bell's observations in the Medico-Chirurgical Transactions.

"If to so powerful a testimony as this my own experience can add any weight, I must ingenuously declare that I have been unsuccessful and disappointed in my expectations whenever I have extirpated the scirrhus, accompanied by unequivocal signs of the tumor having entered into the second stage. In the whole course of a long practice three cases only of extirpation of *genuine* scirrhus of the breast have had a prosperous result with me, these being the only three in which I was so fortunate as to perform the operation within the first months from the appearance of the disease, before the unpleasant sense of pruritus and heat had taken place, and consequently that of the lancinating pains. The operation, in all these instances, was performed by the removal of the whole mammary gland, although the scirrhus occupied scarcely two-thirds of it; and in each of them, when the internal, altered texture of the indurated gland was examined attentively, I found the peculiar and distinctive characters of the legitimate scirrhus. I have effected a cure in a much greater proportion than this, in the excision of the legitimate scirrhus of the testicle; a difference of success which I can attribute to no other cause than the more frequent opportunity of extirpating the scirrhus testicle during the first stage, than that of amputation of the scirrhus of the breast before it has advanced to the state of occult cancer. In two cases I have extirpated the malignant tubercle of the tongue; in the one the cure was compleat and permanent, the darting pains not having yet commenced in it, and much less the appearance of fissures; in the last case, as it was operated on during the period of degeneration, the cancerous sore returned more formidably than before. In one instance only have I been successful in the excision of the *encanthis* indurated by scirrhus of a malignant nature, but indolent, recent, and in the first period of it; the other cases of this sort, from the operation having been performed during the second stage of the disease, had an unsuccessful termination. For the same reason the excision of the parotid and maxillary glands was unsuccessful in those cases which have come within my knowledge, in which the darting pains were unusually violent." 47.

Professor Scarpa feels himself warranted, however, to make exceptions here in favour of warts and hard malignant tubercles of the skin of the lips, nose, and face—maladies which have received the forbidding name of noli me tangere. These seem to our author of a less virulent nature than that of the glandular scirrhus, or even of the malignant tubercles of the reflected skin.

We have now presented our surgical brethren with the sentiments of the distinguished Pavian professor, and we do not deem it necessary to offer any commentaries of our own. We have only now to give an account of a case of sanguineous tumour of the breast, related by Mr. Briggs, in the Appendix of notes to the translation. As the case is short, and admits not of abbreviation, we shall insert it entire.

"1810, *October.*—Mrs. Allardice, a single woman, aged forty-five, a patient of the Public Dispensary, had a tumor in the left breast, extending from within a short distance of the nipple towards the axilla, which originated, as she stated, about eighteen months before, like a small moveable kernel under the skin, sometimes painful when the arm pressed upon it during sleep. Within the last three weeks a fluctuation had been perceptible in it, and it had been judged by the late Mr. Crowther, to be of a cancerous nature.

"30.—The tumour was punctured with the point of a lancet, and about four ounces of a dark and inodorous fluid was discharged from it in a jet, resembling in all respects, except the circumstance of its not coagulating spontaneously, venous blood, and on examination appeared to consist of the serum, with the colouring part of that fluid only.

"On the third day following, the tumor was found to have filled again; a considerable quantity of bloody fluid having flowed from the orifice in the interim, some of which was effused into the cellular membrane, producing ecchymosis of the surrounding skin. The part felt hot and tense, and the pulse quick. From this time until the operation, a period of thirty-one days, the orifice burst open several times, and discharged a large quantity of the same kind of fluid, the tension taken off by this means affording her relief from the pain occasioned by its pressure. The alarm that she felt at the repeated discharges of blood which took place at intervals, and which became more frequent and profuse, induced her to submit to the removal of the diseased part.

"*Dec.* 4.—An elliptical incision was made through the integuments covering the tumor about four inches and a half in extent, and the whole of the indurated part dissected out, laying bare, by this means, a part of the pectoral muscle and the strong fascia covering the *serratus magnus.* Immediately below the edge of the former muscle, running in the same direction, and upon this fascia, a large *venous trunk* was exposed, which bled profusely. Struck with the unusual size of this vessel, it was immediately considered to be the source from which the tumor had originated, as well as the hemorrhage which had taken place subsequently to the puncturing of it. A ligature was accordingly applied upon it, and the lips of the wound brought together in the usual manner.

"Every thing seemed to go on well until the ninth day from the operation, when, to my great disappointment, I found the patient complaining of unusual pain in the wound, from which there was a profuse discharge of a very highly offensive bloody sanious fluid; and on removing the dressings, the lips, particularly towards the axilla, were found to be forced apart by an accumulation of grumous blood.

"Notwithstanding these unfavourable appearances, from which I had apprehensions of the failure of the operation, the sanious bloody discharge which took place from it gradually diminished, and in the course of eight or nine days had entirely ceased. In about six weeks from the time of the operation the parts were perfectly healed.

"This woman continued in an apparently good state of health for eight years afterwards, without any appearance of relapse of the local complaint, and died of sudden illness in St. Bartholomew's Hospital, in the autumn of 1818.

"In the centre of the tumor, when examined, a cavity was found capable of holding three or four ounces of fluid, the inner surface of which presented a number of rugæ or irregular folds, not unlike those of the corrugated inner surface of the bladder, or the auricles of the heart, forming small interstitial cells or foveolæ, communicating with the principal cavity; the entire parieties of the cavity, which were about one-third of an inch in thickness, as well as the small cells formed in the sides of it, were found to consist of depositions of successive layers* of coagulable lymph. A small portion of the glandular part of the breast had undergone some change in its texture, but had no communication with the cavity before-mentioned, and probably arose from pressure made upon it in consequence of its contiguity with the principal diseased part.

"From this account it is evident that the disease originated in the *vascular* and not the *glandular* part of the breast; that the bloody fluid contained in the sac was derived, in some manner or other, from the enlarged *venous* trunk before-mentioned, although the mode of communication cannot be demonstrated, no opening being found in the sac, as far as I could discover, similar to what is met with in aneurismal tumours or ruptures of the vein. It is reasonable, also, to conclude that the obliteration of this vein by the operation put a stop to the further progress of the disease. The formation of the sac by layers of coagulable lymph, and the want of this property in the fluid contained within it, appear to admit of an easy explanation, upon the supposition of the extravasation and lodgment of blood for some time in the breast, and the consequent spontaneous separation of its constituent parts.

"In all the leading circumstances this case will be found to bear a strong resemblance to the histories of 'Collections of Bloody Lymph in the Breast,' recorded by Dr. Monro, with which it ought to be compared as well as that related by Richter, which, though in some respects different, appears, in my opinion, to have been of the same character." 58.

The translation, as our readers will have perceived, is couched in easy and perspicuous language by Mr. Briggs, and we have no doubt is a faithful picture of the original.

---

* "A tumor in the breast, in which there is a cavity similar to this, is preserved in the Museum of the College of Surgeons, but unfortunately without any accompanying history to explain it."

